# Decoherence of interacting Majorana modes

**DOI:** 10.1038/srep12530

**Published:** 2015-07-27

**Authors:** H. T. Ng

**Affiliations:** 1 Center for Quantum Information, Institute for Interdisciplinary Information Sciences, Tsinghua University, Beijing 100084, P. R. China

## Abstract

We study the decoherence of Majorana modes of a fermion chain, where the fermions interact with their nearest neighbours. We investigate the effect of dissipation and dephasing on the Majorana modes of a fermionic chain. The dissipative and dephasing noises induce the non-parity- and parity-preserving transitions between the eigenstates of the system, respectively. Therefore, these two types of noises lead to the different decoherence mechanisms. In each type of noise, we discuss the low- and high-frequency regimes to describe the different environments. We numerically calculate the dissipation and dephasing rates in the presence of long-range interactions. We find that the decoherence rate of interacting Majorana modes is different to that of non-interacting modes. We show the examples that the long-range interactions can reduce the decoherence rate. It is advantageous to the potential applications of quantum information processing.

Majorana fermions are exotic particles^1^ which show non-abelian statistics^2^,^3^,^4^. Indeed, non-abelian statistics is necessary for performing topological quantum computation^5^ which is a kind of fault-tolerant quantum computation. Thus, the study of Majorana fermions is of fundamental importance and also it is useful to the applications of quantum information processing (QIP).

Kitaev predicted that an unbound pair of Majorana fermions^6^ exhibits at the two ends of a spin-polarized one-dimensional (1D) superconductor. This provides a promising way to realize Majorana fermions. Recently, a number of methods has been proposed to simulate Majorana fermions in a 1D system such as by using a semiconductor nanowire^7^,^8^ and cold atoms in an optical lattice^9^,^10^.

Decoherence severely hinders the performance of QIP applications which rely on quantum coherence^11^. The various approaches have been proposed to combat against decoherence such as quantum error correction^12^,^13^ and dynamical decoupling techniques^14^,^15^, etc. Remarkably, Majorana fermions are robust against local perturbations^16^ due to a large energy gap from the two degenerate ground states. It is believed that they can be exploited without further protection. Still, they suffer from decoherence. Recently, decoherence of Majorana modes has been studied in more detail^17^,^18^,^19^,^20^,^21^,^22^. The noises sources from the different physical settings have also been discussed^19^,^20^,^21^.

In addition, the effects of long-range interactions between fermions on the Majorana modes^23^,^24^,^25^,^26^,^27^,^28^ have recently been studied. The long-range interactions can broaden the range of parameters for exhibiting Majorana fermions^23^,^28^. It is natural to ask the effect of long ranged interactions on decoherence of the Majorana modes. In this paper, we study the decoherence rate of Majorana modes of a chain of spinless fermions in the presence of long-range interactions between fermions. Our study is helpful to understand the relationship between interactions and the decoherence properties in a many-body system.

We study the two typical noises in the system, where they are dissipation and dephasing, respectively. These two types of noises are widely studied in the context of open quantum problems and also they are two main forms of decoherence occurring in quantum computing^29^. Dissipation and dephasing lead to the different decoherence mechanisms of Majorana modes. Dissipation induces the non-parity preserving transitions between the eigenstates of the system while dephasing gives rise to parity preserving transitions.

Moreover, we investigate the low- and high-frequency noises to describe the different types of environment. The frequency domain of the low-frequency noise spectrum is much lower than the transition frequency of the two degenerate ground states and their first excited states. For example, the low-frequency noise can be described by the 1/*f*-noise^30^ which commonly occurs in the solid-state devices. On the other hand, the high-frequency noise is to describe the environment in which the frequency domain of the noise spectrum is comparable to the transition frequencies between the different eigenstates. We consider the high-frequency baths to be Markovian in this paper.

We show the examples that the long ranged interactions between fermions can reduce the decoherence rates. In fact, the dissipation and dephasing rates depend on the collective properties of fermions which can be changed by the interactions between the fermions. As a result, *long ranged interactions can change the decoherence properties of Majorana modes*. In this way, the coherence time of the Majorana modes can be prolonged by appropriately choosing the interaction parameters. It may be useful for Majorana-based applications^4^,^5^,^31^.

## System

Majorana modes occur in a spin-polarized 1D superconductor^6^. This 1D superconductor can be described by a chain of spinless fermions with an open boundary condition. The Hamiltonian of this fermionic system is given by, (*ħ* = 1),


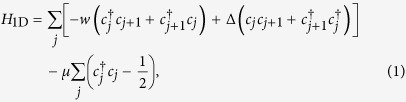


where *c*_*j*_ and 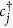 are annihilation and creation fermionic operators at site *j*. The parameters *w*, Δ and *μ* are the tunneling strength, superconducting gap and chemical potential, respectively.

We consider the fermions to be interacted with their nearest neighbors. The Hamiltonian, describes long-range interaction^24^, is written as,


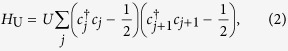


where *U* is the repulsive interaction strength between the nearest neighbours.

A fermionic chain can be mapped onto a spin chain by applying the Jordan-Wigner transformation^16^. The fermionic operators are related to spin-half operators via the Jordan-Wigner transformation as follows:


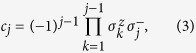



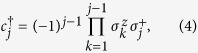



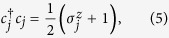


where 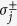 and 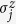 are the Pauli spin operators at site *j*. The Hamiltonian *H* = *H*_1D_ + *H*_U_ of the system can be recast as


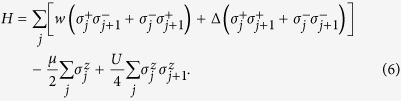


The quantum simulation of the Ising spin chain with the transverse field by using trapped ions has recently been proposed^32^.

This 1D system possesses the 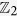 symmetry. The parity operator *P* can be defined as 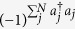 and 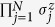 for a fermionic chain and a spin chain, respectively. Therefore, each eigenstate has a definite parity. It is either to be *P* = 1 (even) or *P* = −1 (odd).

## Majorana fermions

Majorana operators can be defined as^6^,^16^


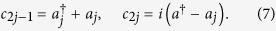


The Majorana operators satisfy the anti-commutation rules, and also they are Hermitian operators. In fact, the Hamiltonian of a fermonic chain can be expressed in terms of Majorana operators^6^,^16^. A pair of unbound Majorana fermions exhibit at the ends of a chain and the remaining Majorana fermions are bounded in pair^6^,^16^. The pair of unbound Majorana fermions (Majorana modes) are shown when the system has the two-fold ground-state degeneracy, where the two degenerate ground states have the different parities.

The Majorana modes can exhibit even if the fermions interact with their nearest neighbours^23^,^28^. This can be indicated by examining the ground-state degeneracy. We calculate the energy difference between the two ground states with the different parities. It can be defined as^23^


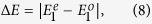


where 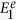 and 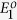 are the ground-state eigen-energies in the even- and odd-parities, respectively. If Δ*E* is zero, then the system supports the Majorana modes^23^.

**Figure 1:**
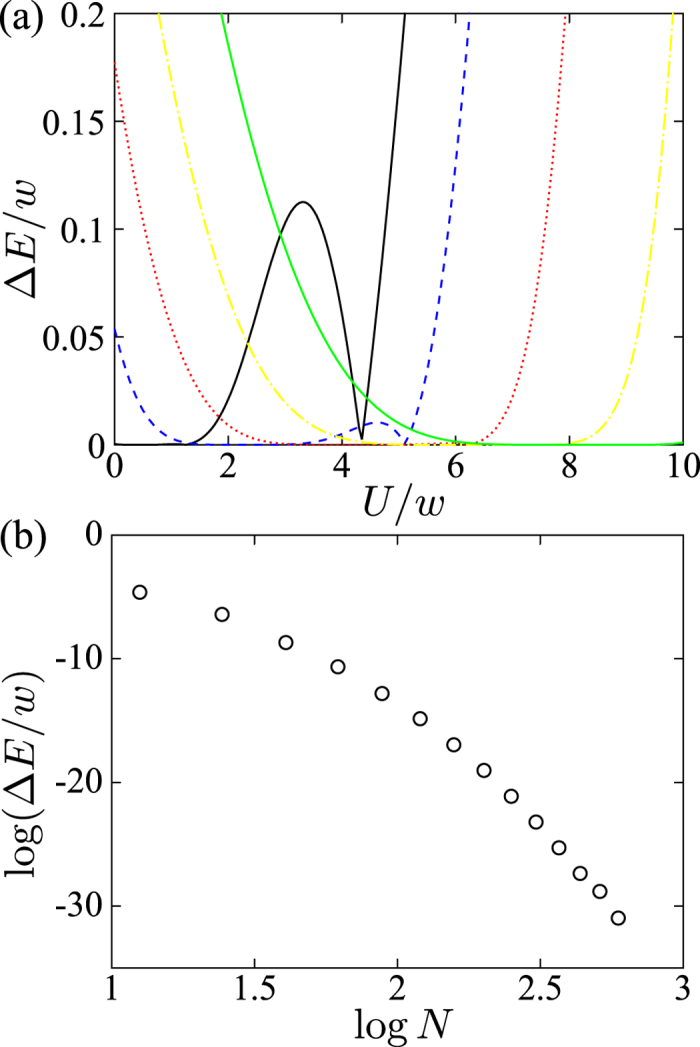
In (**a**) energy gap Δ*E* versus interaction strength *U*, for *N* = 12 and *μ* = *w*. The different interaction strengths Δ are denoted by the different lines: Δ = *w* (black solid), 2*w* (blue dashed), 3*w* (red dotted), 4*w* (yellow dash-dotted) and 5*w* (green solid), respectively. In (**b**) log-log plot of energy gap Δ*E* versus *N*, for *μ* = *w*, Δ = 5*w* and *U* = 8*w*.

We numerically solve the Hamiltonian in Eq. (6) by using exact diagonalization. In Fig. 1(a), we plot the energy difference Δ*E* as a function of interaction strength *U*, for the different interaction strengths Δ. The zero energy gap is shown, this implies that the Majorana modes exist. When Δ increases, the broader range of interaction strength *U* can be obtained. We also study the relation of the energy gap and the size *N* of system. In Fig. 1(b), we plot Δ*E* verus *N* in the logarithmic scale. The energy gap exponentially decreases as the size *N*. This shows that the feature of topological degeneracy^16^.

## Phase diagram

To understand the ground-state properties of the system, we briefly discuss the phase diagram. To facilitate our discussion, we recast the Hamiltonian in Eq. (6) as


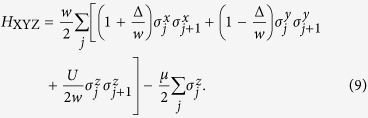


Indeed, it is the *XYZ* model^25^,^33^. Note that the system is invariant if the sign of *μ* is changed, i.e., *μ* → −*μ*. This can be seen by transforming the spin operators 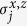 into 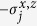. The Hamiltonian *H*_XYZ_ in Eq. (9) remains unchanged.

**Figure 2:**
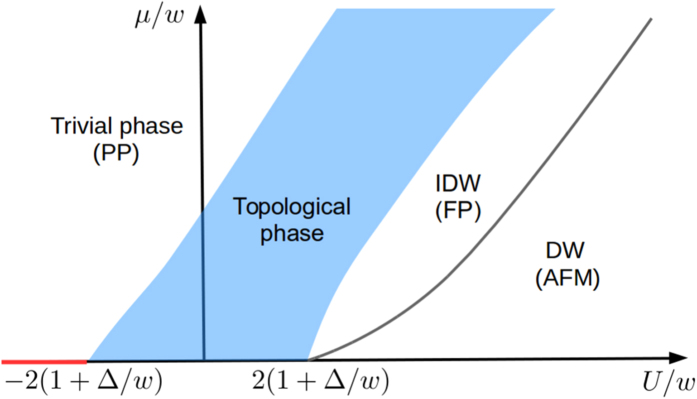
Schematic of phase diagram of the *XYZ* model (see, e.g.^25^,^26^,^27^,^33^). The red line is marked for the transition when *μ* = 0 and *U* < −2(1 + |Δ|/*w*)*w*.

The phase diagram of the *XYZ* model is known^25^,^26^,^27^,^33^. Let us briefly discuss their results. The schematic of phase diagram as a function of *μ* and *U* is shown in Fig. 2. This system has the four different phases. They are trivial, topological, density-wave (DW) and incommensurate density-wave (IDW) phases. The topological phase can be found by examining the energy difference Δ*E* in Eq. (8) between the two ground states with the different parities^23^,^27^. The DW and IDW phases can be found when the two ground states occur in the same parity^27^. The DW phase is also called the anti-ferromagnetic (AFM) in which the total magnetization becomes zero in the *z* direction^33^. But the IDW phase, which is termed as floating phase^25^,^26^,^33^, has a finite magnetization. Also, at the zero magnetic field (*μ* = 0), the system is characterized by a ferromagnetic (FM) phase^33^ for large negative *U*. When the magnetic field becomes large, the system is in a trivial (PP) phase with a large magnetization which depends on the direction of the magnetic field. There is a transition^33^ between them when *U* is less than −2(1 + |Δ|/*w*)*w*.

**Figure 3:**
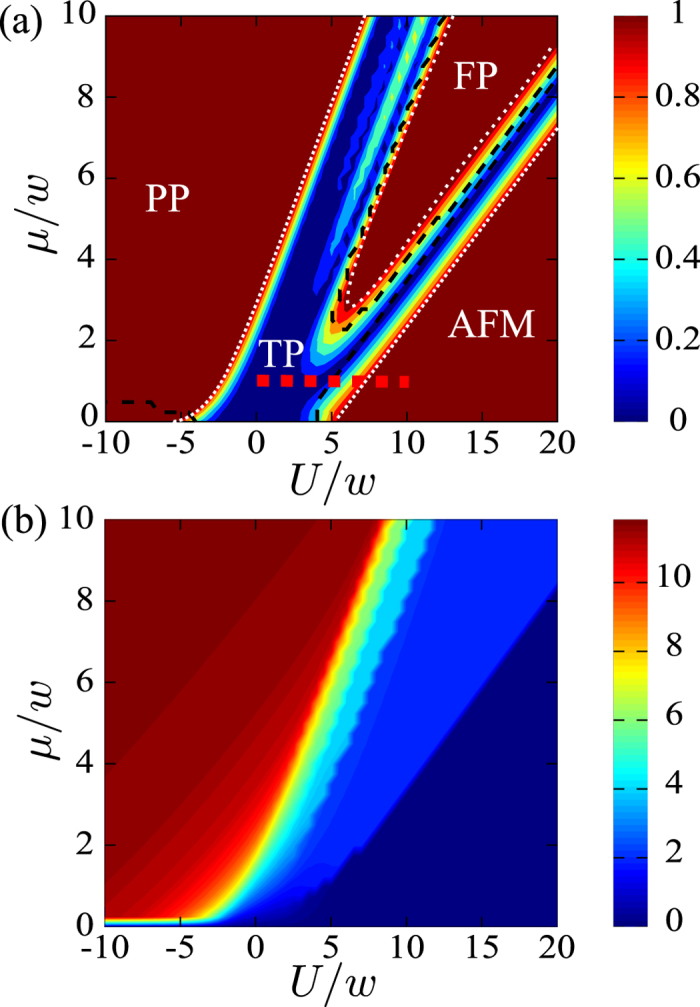
Contour plots of Δ*E* and *M* versus interaction strengths *μ* and *U* in (a,b) respectively, for *N* = 12 and Δ = *w*. In (**a**) the black dashed lines are marked to indicate that the two ground states occur in the same parity. The red horizontal dotted line is marked for the parameters we discussed in the subsequent figures. The different phases are labelled and the white dotted lines are used for showing the phase region.

We examine the “finite-size” phase diagram by studying Δ*E* and the total magnetization 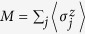 in the *z* direction. In Fig. 3(a), the contour plot of Δ*E* is plotted as a function of *μ* and *U*. The topological phase (TP) can be indicated when Δ*E* = 0, i.e. the deep blue region in Fig. 3(a). Indeed, the topological phase can be described by the two Néel states in the *x*-direction. A more detailed discussion can be found in supplementary information. When the two ground states occur in the same parity, the DW and IDW phases can be distinguished from the topological phase in Fig. 3(a). Also, the transition between the FM and PP phases at zero *μ* can also be indicated in Fig. 3(a). In addition, we plot the total magnetization *M* versus *μ* and *U* in Fig. 3(b). The trivial (PP) and DW (AFM) phases can be clearly shown. But the transition between the topological phase and IDW phase cannot be distinguished by this method^33^. By comparing the energy gap and its parity and also the magnetization, we are able to determine the phase which is labelled in Fig. 3(a). The transitions between the different phases cannot be manifestly shown due to the relatively small size of the system.

**Figure 4:**
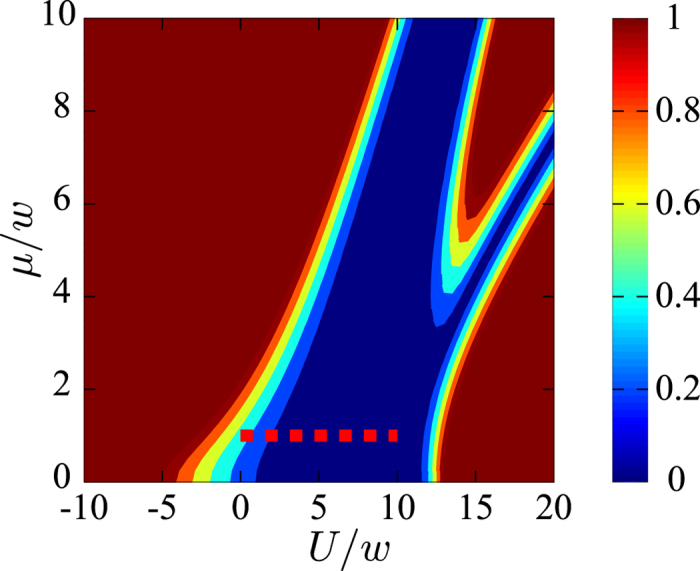
Contour plots of Δ*E* versus interaction strengths *μ* and *U*, for *N* = 12 and Δ = 5*w*. The red dotted horizontal line is marked for the parameters used in the subsequent figures.

In Fig. 4, we show the contour plot of Δ*E* versus *μ* and *U* with a larger Δ = 5*w*. In this case, the region of nearly zero Δ*E* becomes larger than that in Fig. 3(a) since *U* increases. This means that the topological phase can be obtained with a wider range of parameters. However, the topological phase tends to shift to the right-hand side and it is smaller than that of the schematic phase diagram in Fig. 2 due to the finite-size effect.

## Decoherence

We consider the fermions to be coupled to an environment. This causes decoherence of the Majorana modes. We study the two different types of noises which are dissipation and dephasing, respectively.

In general, the total Hamiltonian, which includes the system and bath and their interactions, can be written as


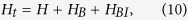


where *H*, *H*_*B*_ and *H*_*BI*_ are the Hamiltonians of the system, bath and system-bath interactions, respectively. It is convenient to express the Hamiltonian *H*_*t*_ in terms of the system’s eigenstates, i.e.,


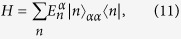


where 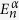 is the eigen-energy of the *n*-th eigenstate |*n*〉_*α*_ of the system in the even (*α* = *e*) and odd (*α* = *o*) parities. In the interaction picture, the Hamiltonian *H*_*BI*_ can be written in terms of the eigenstate |*n*〉_*α*_ as


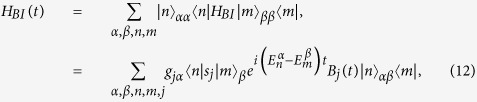


where *g*_*j*_ is the system-bath coupling strength, *s*_*j*_ and *B*_*j*_(*t*) are the system and bath operators at site *j*, and *α*, *β* = *e* and *o*. Here we study the eigenstates of a spin chain which can be easier to numerically implement.

For the low-frequency noise, we consider the frequency domain of the noise spectrum to be much lower than the transition frequency between the degenerate ground states and their first excited states. However, the two degenerate ground states are still subject to low-frequency noise.

In the case of high-frequency noise, the frequency domain of the noise spectrum is comparable to the transition frequencies between the different eigenstates. We assume that the coupling between the system and bath is weak so that the Born-Markovian approximation can be applied. At zero temperature, the system maintains in the two degenerate ground states. We have also assumed that the coupling between the two degenerate ground states and the bath is zero for this environment. However, the bath will induce the transitions between the degenerate ground states and higher excited states at finite temperature. In the subsequent discussion, we will study the low- and high-frequency regimes in the different types of noises.

### Dissipation

**Figure 5:**
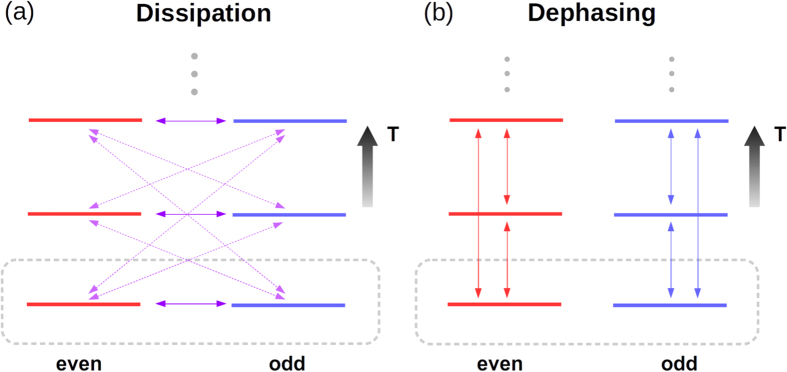
Transitions between eigenstates via dissipation in (a) and dephasing in (b). In (**a**) dissipation induces the transitions between the eigenstates with the different parities. In (**b**) dephasing induces the transitions in the same parity. In both cases, transitions between the two degenerate states occur via low-frequency noise, and transitions between higher excited states occur through high-frequency noise at finite temperature.

In this subsection, we discuss the effect of dissipation on the Majorana modes. The Hamiltonian of system-bath interaction, which describes the dissipation, is of the form:


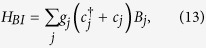


where *g*_*j*_ and *B*_*j*_ are the system-bath coupling strength and the bath operator, respectively. Here each fermion independently couples to a fermionic bath. Such dissipation noise leads to transitions between the eigenstates in the different parities. Transitions between the eigenstates in the different parities is shown in Fig. 5(a).

#### Low-frequency noise

Here we consider the low-frequency noise to be dominant. The frequency domain of the noise spectrum is much lower than the transition frequency between the two degenerate ground states and their first excited states. The Hamiltonian, describes the interaction between the two degenerate ground states and the bath, can be written as


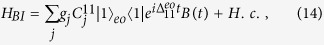


where 
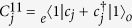
, 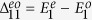, and *B*(*t*) is a time-dependent bath operator. Here 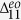 is very close to zero. It should be noted that the the dissipation does not cause the energy damping to the two ground states in the low-frequency noise, but it leads to decoherence.

We assume that the system-bath coupling strengths *g*_*l*_ ≈ *g* are nearly equal. The coupling strength between the two ground states and the bath is given by


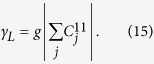


**Figure 6:**
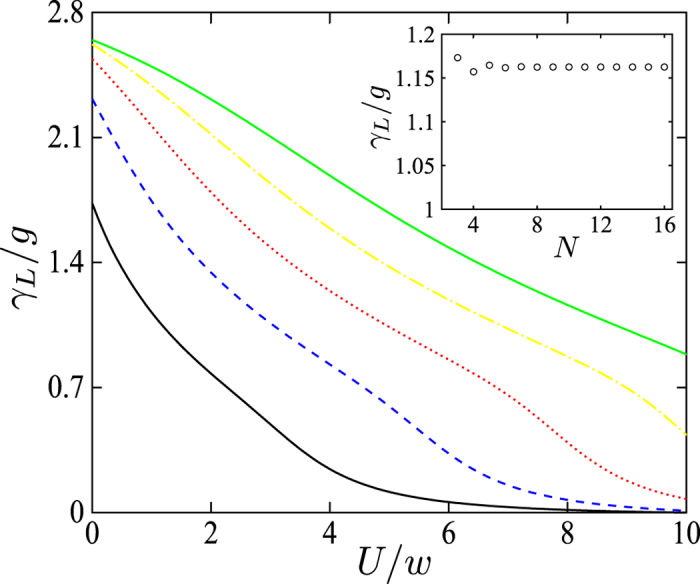
Parameter *γ*_*L*_ versus interaction strength *U*, for *N* = 12 and *μ* = *w*. The different interaction strengths Δ are denoted by the different lines: Δ = *w* (black solid), 2*w* (blue dashed), 3*w* (red dotted), 4*w* (yellow dash-dotted) and 5*w* (green solid), respectively. In the inset, the parameter *γ*_*L*_ versus *N*, for *μ* = *w*, Δ = 5*w* and *U* = 8*w*.

The decoherence rate is closely related to the parameter *γ*_*L*_. In fact, the decoherence rate also depends on the explicit property of the noise spectrum^30^. For example, we consider 1/*f* noise which can be described by the spin fluctuator model. The decoherence rate is proportional to the ratio of *γ*_*L*_ to *γ*_*f*_, where *γ*_*f*_ is the switching rate of spin fluctuator. Therefore, the parameter *γ*_*L*_ plays an important role to describe the decoherence effect. Here we investigate the parameter *γ*_*L*_ only. This parameter *γ*_*L*_ can reflect how strong the decoherence effect is. In Fig. 6, we plot *γ*_*L*_ versus the interaction strength *U*, for the different strengths Δ. The parameter *γ*_*L*_ decreases as *U* increases. This means that the interactions between fermions can reduce the decoherence rate in the low-frequency regime. In addition, we plot *γ*_*L*_ versus *N* in the inset of Fig. 6. The parameter *γ*_*L*_ is nearly constant when the system *N* grows. We briefly discuss why this parameter *γ*_*L*_ does not depend on *N* in supplementary information.

#### High-frequency noise

Now we study the effect of dissipation on the Majorana modes, where the frequency domain of the noise spectrum is comparable to the transition frequencies between the different eigenstates. We assume that this high-frequency noise does not affect the dynamics between the two degenerate ground states, where their transition frequency is nearly zero. We consider that the environment can be modelled by a bath of fermions. In the interaction picture, the Hamiltonian of system-bath coupling can be written as


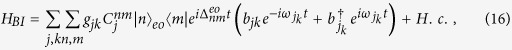


where 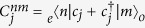 and 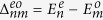. The coupling strength 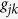 is much smaller than 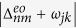, where 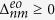 and *n* > *m*. Therefore, we can apply the rotating-wave-approximation (RWA) to ignore the fast-oscillating terms. The Hamiltonian can be written as


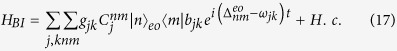


We assume that the Born-Markovian approximation can be applied to this system. The master equation can be derived^34^ in the dressed-state picture which can provide the correct steady state even for a strongly interacting system. The master equation, which describes the dissipation, can be written as^34^


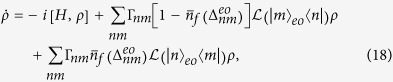


where 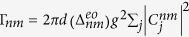 and 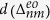 is the density of states, 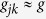 and *n* > *m*. The parameter 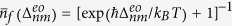 is the mean occupation number for fermions at the frequency 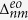, where *k*_*B*_ is the Boltzmann constant and *T* is the temperature. The superoperator 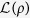 is of the Lindblad form as^35^


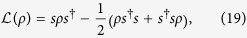


where 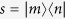 and *m* < *n*.

The master equation in Eq. (18) is valid if there is no degeneracy between the transitions^34^. We assume that there is no degeneracy between the transitions in deriving the master equation in Eq. (18). The energy difference 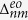 is large enough and the system-bath coupling 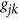 is sufficiently weak. Therefore, the RWA can be applied to the master equation to ignore the fast-oscillating terms^34^. Although it may encounter the accidental degeneracy of the transitions between the higher excited states, we can ignore those transitions within the coherence time of the degenerate ground states at low temperature. The master equation can give a reasonably good approximation to describe the dynamics of the Majorana modes.

**Figure 7:**
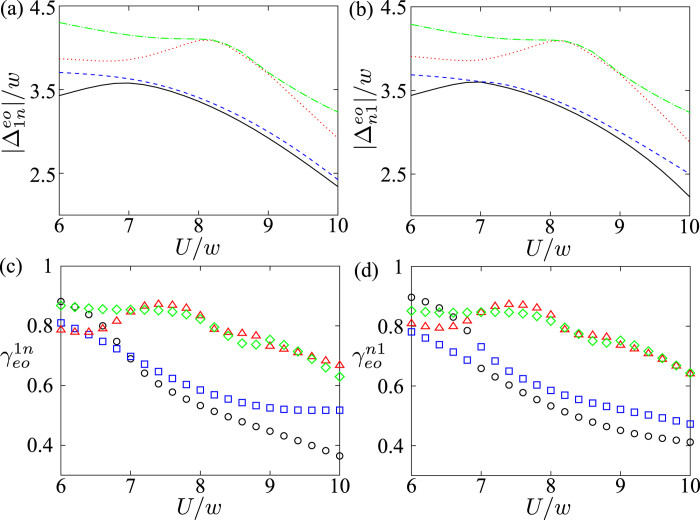
Energy differences versus *U* in(a,b). The energy differences 
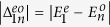
 and 
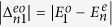
 are plotted in (**a**,**b**) respectively. The different transitions *n* are denoted by the different lines: *n* = 2 (black solid), 3 (blue dashed), 4 (red dotted) and 5 (green dot-dash), respectively. Parameters 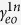 and 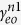 are plotted versus *U* in (**c**,**d**). The different transitions *n* are denoted by the different symbols: *n* = 2 (black circle), 3 (blue square), 4 (red upper triangle) and 5 (green diamond), respectively. Parameters are used: *N* = 12, *μ* = *w* and Δ = 5*w*.

In Fig. 7(a,b), we plot the energy differences, 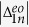 and 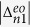, between the ground states and the first four eigen-energies in their opposite parities, respectively. The energy difference decreases when the system exhibits the Majorana fermions, i.e., Δ*E* = 0 for Δ = 5*w* in Fig. 1. Therefore, the mean number 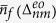 increases. Also, it should be noted that the degeneracy between the higher excited states occurs as shown in Fig. 7(a,b). This master equation can still be used to describe the dissipative dynamics in the wide range of parameters except those degeneracy points.

The dissipation rate Γ_*nm*_ is proportional to 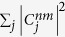. Let us denote the parameters 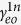 and 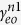 to be 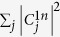 and 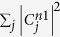, respectively. These parameters give the transition rates between the ground state and higher excited states in the opposite parity. In Fig. 7(c,d), we plot the parameters 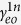 and 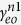 versus *U*, where *n* = 2, 3, 4 and 5. These two parameters decreases when *U* increases. Thus, the dissipation rates Γ_1*n*_ and Γ_*n*1_ also decrease. We can see that the interchange of the parameters 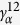 and 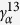 occurs around *U* = 7*w* in Fig. 7(c,d). It is because the two energy levels avoid crossing around *U* = 7*w* in Fig. 7(a,b), and the wavefunction must be continuous at this point. Although the mean number 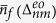 increases as *U* increases, the parameters 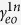 and 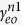 decreases. Therefore, 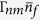 decreases if the temperature *T* is sufficiently low. The interaction between fermions can reduce the effect of dissipation at low temperature.

**Figure 8:**
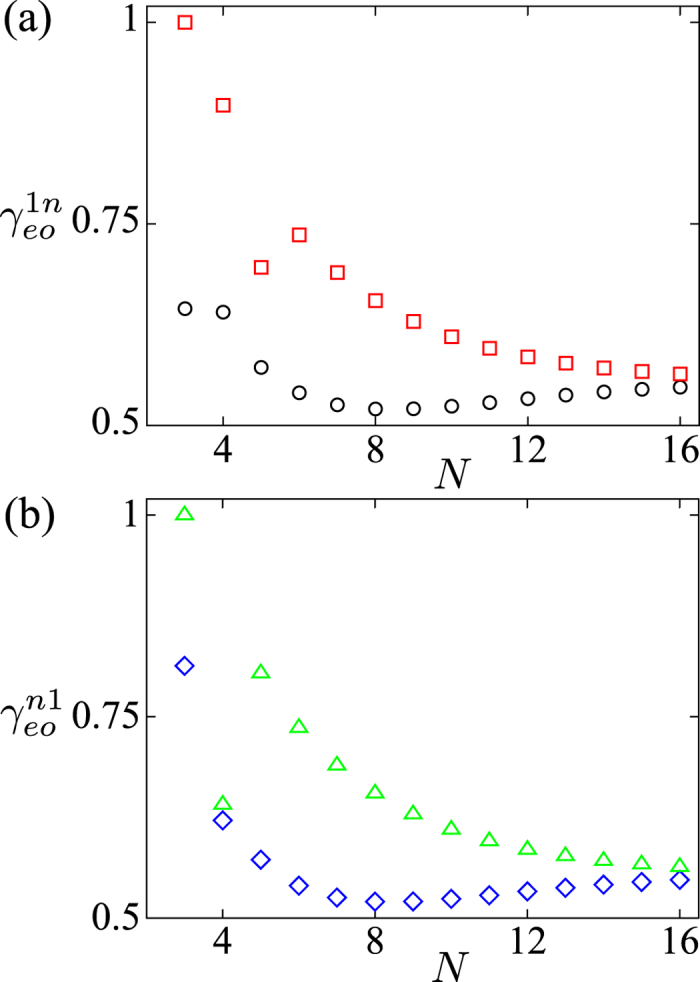
Parameters 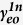 and 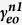 versus *N*, for *n* = 2,3. In (**a**) 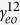 and 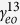 are denoted by black circle and red square, respectively. In (**b**) 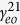 and 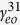 are denoted by blue diamond and green upper triangle, respectively. The parameters are used: *μ* = *w*, Δ = 5*w* and *U* = 8*w*.

Also, we study the relationship between the behaviours of 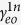 and 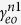 and the system’s size. In Fig. 8, we plot the two parameters 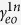 and 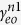 versus *N*, for *n* = 2, 3. The parameters 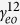 and 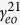 decreases with small *N*, and then slightly increases when *N* becomes larger. The parameters 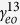 and 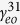 decrease with *N*. Besides, the parameters 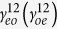 and 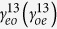 start to converge at *N* = 16 in Fig. 8.

### Dephasing

We study the effect of dephasing on the Majorana modes. In contrast to the case of dissipation, the dephasing noise gives rise to the transitions between the eigenstates in the same parity. In this model, the fermions are coupled to a common bosonic bath. The Hamiltonian, describes the system-bath coupling, is given by


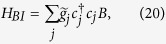


where 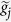 is the coupling strength at site *j* and *B* is the bath operator. This decoherence model is similar to the model discussed in^19^. Dephasing can induce the transitions between the eigenstates of the system which are summarized in Fig. 5(b).

#### Low-frequency noise

**Figure 9:**
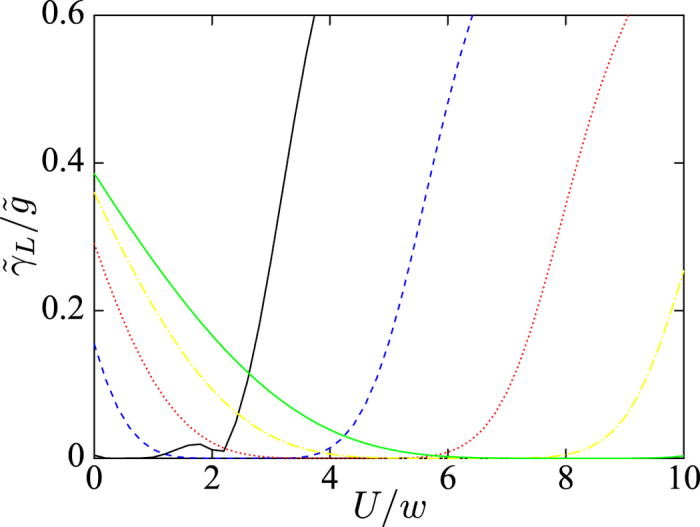
Parameter 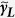 versus interaction strength *U*, for *N* = 12 and *μ* = *w*. The different interaction strengths Δ are denoted by the different lines: Δ = *w* (black solid), 2*w* (blue dashed), 3*w* (red dotted), 4*w* (yellow dash-dotted) and 5*w* (green solid), respectively.

We study the effect of dephasing in the low-frequency regime. In this regime, we can express this Hamiltonian in terms of eigenstates of the two lowest degenerate states. Now the Hamiltonian is given by


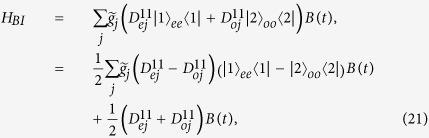


where 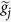 is the coupling strength, 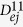 and 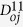 are 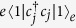 and 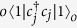, respectively. The effective coupling strength between the Majorana modes and bath is


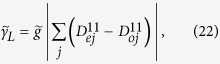


where 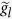 is roughly equal to 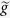. We study the relationship between the coupling strength 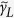 and the interaction strength *U*. In Fig. 9, we plot the parameter 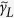 versus *U*, for the different strengths Δ. The numerical results show that 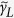 can reach nearly zero when the Majorana modes exhibit (Δ*E* = 0 in Fig. 1). This shows that Majorana modes are robust against the low-frequency dephasing noise. In fact, this can be easily understood by writing the fermion operator in terms of spin operators. From Eq. (5), we have 
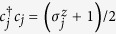
. It will flip the spin state from 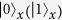 to 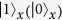. It gives 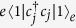 and 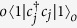 to be 0.5 if the two degenerate ground states can be approximately described by the two Néel states. Therefore, the parameter 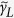 is nearly zero.

#### High-frequency noise

We consider the frequency domain of the noise spectrum to be comparable to the transition frequency between the different eigenstates. We presume that the high-frequency noise will not affect the dynamics between the two degenerate ground states. We follow the similar treatment in the previous subsection to study the high-frequency noise. We assume that the coupling between the system and bosonic bath is sufficiently weak, so that the RWA can be applied. In the interaction picture, the Hamiltonian of system-bath coupling can be approximated as


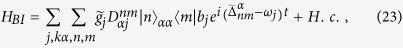


where 
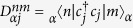
 and 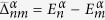, *α* = *e*, *o*. Here the energy difference 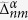 is positive and *n* > *m*.

The master equation can be obtained by using the Born-Markovian approximation^34^. The master equation, describes the dephasing noise, can be written as


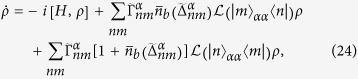


where 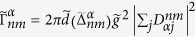, 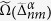 is the density of states, 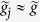 and *n* > *m*. The parameter 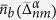 is the mean occupation number, for the bosons, at the frequency 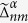 and the temperature *T*. Here we have assumed that there is no degeneracy in the transitions^34^.

**Figure 10:**
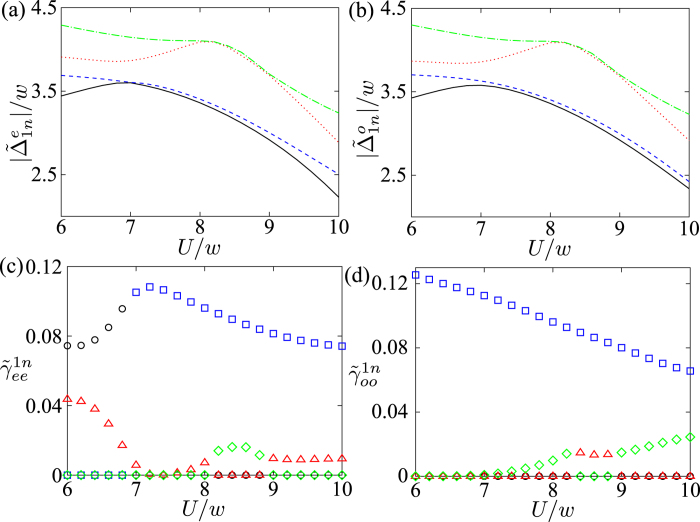
Energy differences versus *U* in (a,b). The energy differences 
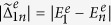
 and 
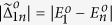
 are plotted in (**a**,**b**), respectively. The different transitions *n* are denoted by the different lines: *n* = 2 (black solid), 3 (blue dashed), 4 (red dotted) and 5 (green dot-dash), respectively. In (**c**,**d**), the parameters 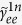 and 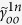 are plotted versus *U*. The different transitions *n* are denoted by the different lines: *n* = 2 (black circle), 3 (blue square), 4 (red upper triangle) and 5 (green diamond), respectively. Parameters are used: *N* = 12, *μ* = *w* and Δ = 5*w*.

In Fig. 10(a,b), we plot the energy differences 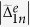 and 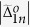 between the ground state and the first four excited states in the same parity. The energy difference decreases when *U* increases. The mean number 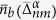 also increases with *U*. Then, we study the parameters 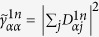 as a function of *U*. They are proportional to the dephasing rate 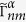. In Fig. 10(c,d), we plot 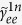 and 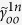 versus *U*, where *n* = 2, 3, 4 and 5. For even-parity transitions, the parameter 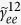 increases and then decreases when *U* attain 7*w*, and 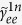 are much smaller than 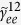 for higher *n*. In the case of odd-parity transitions, the parameters 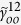 decreases when *U* increases. The parameter 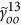 is nearly zero. However, 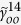 increases as *U* becomes larger. Since the energy difference between the ground state and the third and forth excited states are larger, this transition is less important compared to the other transitions with the smaller energy gaps. The effect of dephasing, 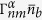, should be small if the temperature is sufficiently low.

**Figure 11:**
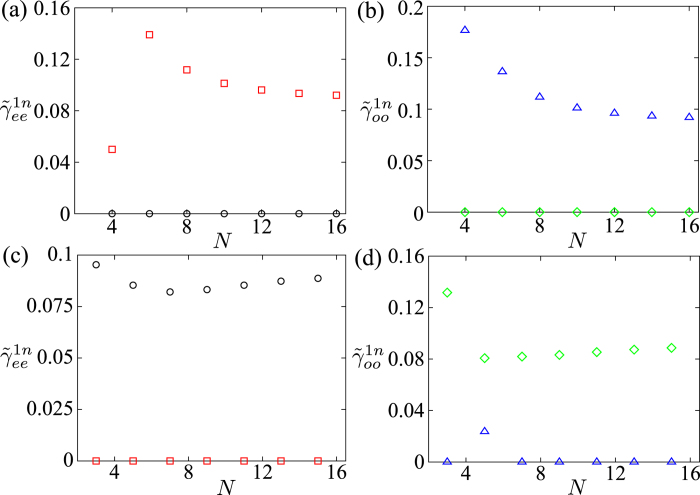
Parameters 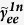 and 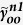 versus *N*, for *n* = 2,3. The even number of fermions are plotted in (**a**,**b**) and the odd number of fermions are plotted in (**c**,**d**). In (**a,c**) 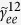 and 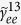 are denoted by black circle and red square, respectively. In (**b**,**d**) 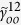 and 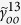 are denoted by green diamond and blue upper triangle, respectively. The parameters are used: *μ* = *w*, Δ = 5*w* and *U* = 8*w*.

We also study the behaviours of the parameters 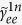 and 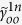, for the different system’s sizes. In Fig. 11, we plot the parameters 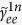 and 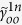 versus *N*. The results are different for the even- and odd-number of fermions. The parameter 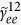 is much smaller(larger) than 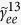 in the even(odd)-number case. Similarly, 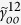 is much smaller(larger) than 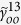 if *N* is even(odd).

## Discussion

We have investigated the two general types of noises which are dissipation and dephasing, respectively. The low- and high-frequency noises are also discussed in each type of noise. Although we have not discussed the noise source for a specific environment, our study should capture the essential feature of the decoherence properties for various types of environment. We show the examples that long-range interactions between the fermions can change the decoherence properties of the Majorana modes. This is the main result of our paper.

In addition, our study is related to the fundamental problem in quantum mechanics. It is an important question on the validity of quantum mechanics in the macroscopic regime^36^,^37^. Indeed, studies of macroscopic superpositions^38^ shed light on this fundamental question^37^. One can consider to create a superpositions of the two degenerate ground states of a fermonic chain which can be realized by either a 1D topological superconductor^6^ or trapped-ion chain^32^. Although it is impossible to create the superposition states of two Majorana fermions of a single chain^17^,^21^,^31^ according to the superselection rule, it can be resolved by encoding the states by using the four Majorana fermions with two fermionic chains. We assume that decoherence does not set in between the two chains. Our present analysis can then be directly applied to this case. For a spin chain, the superposition of two degenerate ground states can be created. The similar study can also be done. In fact, the fermionic and spin chains can be regarded as macroscopic systems. Thus, the decoherence properties of Majorana modes is important to understand the behavior of such superposition states.

## Conclusion

In summary, we have studied the effect of dissipation and dephasing on the Majorana modes of a fermionic chain in the presence of the nearest neighbor interactions between the fermions. The dissipation and dephasing noises can induce the parity- and non-parity preserving transitions. We have also investigated the low- and high-frequency noises to describe the different kinds of environment. We show the examples that the dissipation and dephasing rates can be reduced by increasing the interaction strength at the sufficiently low temperature. This means that the coherence time of Majorana fermions can be extended. It may be useful to the applications of QIP. In addition, we have studied the relationship between the decoherence rate and the system’s size.

## Additional Information

**How to cite this article**: Ng, H. T. Decoherence of interacting Majorana modes. *Sci. Rep*. **5**, 12530; doi: 10.1038/srep12530 (2015).

## Supplementary Material

Supplementary Information
