# Supplementary material for: Decoherence of interacting Majorana modes
Source: Sci Rep. 2015 Jul 27;5:12530. doi: 10.1038/srep12530 (PMC4515602; doi:10.1038/srep12530)
Supplement: Supplementary Information [file srep12530-s1.pdf]

Supplementary information
Decoherence of interacting Majorana modes

H. T. Ng*

Center for Quantum Information, Institute for Interdisciplinary Information Sciences, Tsinghua University, Beijing 100084, P. R. China

SPIN-SPIN CORRELATIONS

The topological phase can be approximately described by the two Néel states in the x-direction, i.e., $|1010\ldots10\rangle_x$ and $|0101\ldots01\rangle_x$. The properties of the topological phase can be manifested by studying spin-spin correlations $\langle\sigma_i^\alpha\sigma_j^\alpha\rangle$, where $\alpha = x, y$ and z. In Fig. S1, we plot the spin-spin correlations $\langle\sigma_1^\alpha\sigma_j^\alpha\rangle$ of the ground state versus site j, for the different strengths Δ. When $\Delta = w$, spin-spin correlations $\langle\sigma_1^x\sigma_j^x\rangle$ alternate positive and negative ones with their neighbouring spins in Fig. S1(a), and also they are about constant as the distance $|1-j|$ increases. The magnitude of correlations $\langle\sigma_1^x\sigma_j^x\rangle$ are larger than the other correlations $\langle\sigma_1^y\sigma_j^y\rangle$ and $\langle\sigma_1^z\sigma_j^z\rangle$. This shows that the topological phase can be approximately described by the two Néel states in the x-direction. As Δ increases, the interaction terms $\sigma_i^y\sigma_{i+1}^y$ appear. In Figs. S1(b) and (c), the spin-spin correlations $\langle\sigma_1^x\sigma_j^x\rangle$ are still dominant and show alternating positive and negative numbers with their neighbours. However, the correlations decays as the distance $|1-j|$ increases. This means that the Néel states are no longer a good approximation to describe the ground state.

We also study the spin-spin correlations of the ground states in the PP and AFM phases, respectively. In Fig. S2, we plot spin-spin correlations $\langle\sigma_1^\alpha\sigma_j^\alpha\rangle$ versus spin j, where the system is in the PP phase. In this case, the spins are polarized. Now $\langle\sigma_1^z\sigma_j^z\rangle$ are nearly one as the distance between the spins increases, and $\langle\sigma_1^z\sigma_j^z\rangle$ are much larger than the other correlations $\langle\sigma_1^x\sigma_j^x\rangle$ and $\langle\sigma_1^y\sigma_j^y\rangle$.

In Fig. S3, we plot $\langle\sigma_1^\alpha\sigma_j^\alpha\rangle$ versus spin j, where the system is in the AFM phase. We can see that the correlations in the z-direction are in alternating positive and negative ones with their neighbours. The other components of correlations are very small. This clearly shows that the ground state is in the AFM phase.

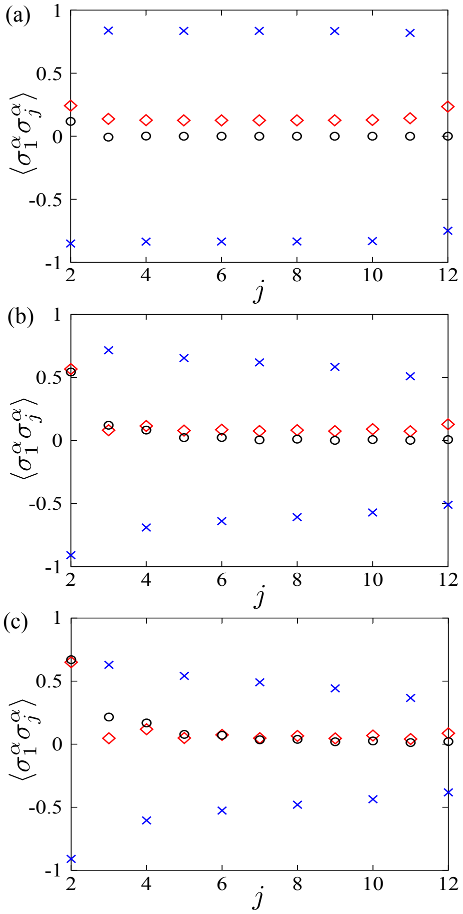

FIG. S1. (Color online) Spin-spin correlations $\langle\sigma_1^\alpha\sigma_j^\alpha\rangle$ versus site j, where $N = 12$, $\mu = w$ and $U = 0$. The different strengths of Δ are plotted: (a) $\Delta = w$, (b) $\Delta = 3w$ and (c) $\Delta = 5w$. The different spin-spin correlations $\langle\sigma_1^\alpha\sigma_j^\alpha\rangle$ are denoted by $\alpha = x$ (blue cross), $\alpha = y$ (black circle) and $\alpha = z$ (red diamond), respectively.

DECOHERENCE RATE OF LOW-FREQUENCY DISSIPATIVE NOISE

For the low-frequency dissipative noise, the decoherence rate is closely related to the parameter γ_L which is given by

$$\gamma_L = g\Big|\sum_j C_j^{11}\Big|, \tag{1}$$

and

$$C_j^{11} = {}_e\langle 1|c_j + c_j^\dagger|1\rangle_o. \tag{2}$$

The numerical result shows that the parameter γ_L does not show the dependence on the system size N. This can be explained by considering the ground state in the topological phase. The two degenerate ground states

* Corresponding author; hotsangng@tsinghua.edu.cn

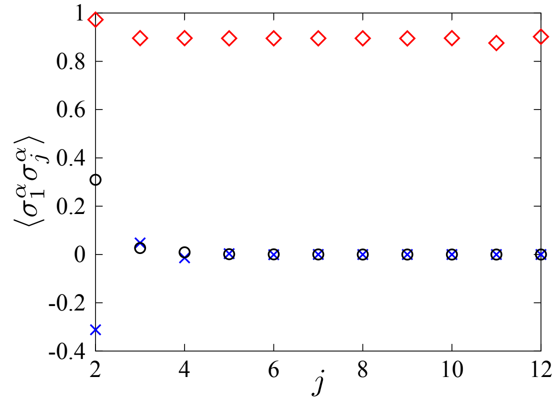

FIG. S2. (Color online) Spin-spin correlations $\langle \sigma_1^\alpha \sigma_j^\alpha \rangle$ versus site j, where $N = 12$, $\mu = 10w$, $\Delta = 5w$ and $U = -20w$. The different spin-spin correlations $\langle \sigma_1^\alpha \sigma_j^\alpha \rangle$ are denoted by $\alpha = x$ (blue cross), $\alpha = y$ (black circle) and $\alpha = z$ (red diamond), respectively.

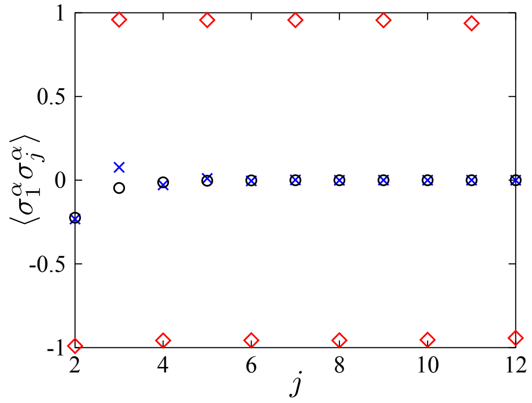

FIG. S3. (Color online) Spin-spin correlations $\langle \sigma_1^\alpha \sigma_j^\alpha \rangle$ versus site j, where $N = 12$, $\mu = w$, $\Delta = 5w$ and $U = 20w$. The different spin-spin correlations $\langle \sigma_1^\alpha \sigma_j^\alpha \rangle$ are denoted by $\alpha = x$ (blue cross), $\alpha = y$ (black circle) and $\alpha = z$ (red diamond), respectively.

can be approximately described by the superposition of two Néel states in the x-direction, i.e., $|1010\ldots10\rangle_x$ and $|0101\ldots01\rangle_x$. The operator $c_j + c_j^\dagger$ can be expressed in terms of the spin-half operators as

$$c_j + c_j^\dagger = (-1)^{j-1} \prod_{k=1}^{j-1} \sigma_k^z (\sigma_j^- + \sigma_j^+). \tag{3}$$

For example, we apply $c_1 + c_1^\dagger$ and $c_2 + c_2^\dagger$ to the state $|1010\ldots10\rangle_x$. We have

$$(c_1 + c_1^\dagger)|1010\ldots10\rangle_x = (\sigma_1^- + \sigma_1^+)|1010\ldots10\rangle_x, \tag{4}$$
$$= |1010\ldots10\rangle_x, \tag{5}$$

and

$$(c_2 + c_2^\dagger)|1010\ldots10\rangle_x, \tag{6}$$
$$= -\sigma_1^z(\sigma_2^- + \sigma_2^+)|1010\ldots10\rangle_x, \tag{7}$$
$$= (-1)(-1)|0010\ldots10\rangle_x, \tag{8}$$
$$= |0010\ldots10\rangle_x \tag{9}$$

The operator σ_k^z will change the state $|0\rangle_x(|1\rangle_x)$ to $|1\rangle_x(|0\rangle_x)$. Similarly, the product of operators $\prod_{k=1}^{j-1} \sigma_k^z$ will change the polarization of spin from 1 to $j-1$. Therefore, we have

$${}_x\langle 1010\ldots10|(c_1 + c_1^\dagger)|1010\ldots10\rangle_x = 1, \tag{10}$$
$${}_x\langle 0101\ldots01|(c_1 + c_1^\dagger)|0101\ldots01\rangle_x = -1 \tag{11}$$

and

$${}_x\langle 1010\ldots10|(c_j + c_j^\dagger)|1010\ldots10\rangle_x = 0, \tag{12}$$
$${}_x\langle 0101\ldots01|(c_j + c_j^\dagger)|0101\ldots01\rangle_x = 0, \tag{13}$$
$${}_x\langle 0101\ldots01|(c_j + c_j^\dagger)|1010\ldots10\rangle_x = 0, \tag{14}$$

for $j \neq 1$. The parameter C_1^{11} is about 1 and $C_{j\neq1}^{11} = 0$ if the ground state can be described by the superposition of two Néel states with equal weights. Thus, the parameter γ is close to g.
